# Predicting Oxidation Potentials with DFT-Driven Machine Learning

**DOI:** 10.1021/acs.jcim.5c00159

**Published:** 2025-05-28

**Authors:** Shweta Sharma, Natan Kaminsky, Kira Radinsky, Lilac Amirav

**Affiliations:** 1 Schulich Faculty of Chemistry, 26747 Technion − Israel Institute of Technology, Haifa 32000, Israel; 2 Taub Faculty of Computer Science, 26747 Technion − Israel Institute of Technology, Haifa 32000, Israel

## Abstract

We introduce OxPot, a comprehensive open-access data set comprising over 15 thousand chemically diverse organic molecules. Leveraging the precision of DFT-derived highest occupied molecular orbital energies (*E*_HOMO_), OxPot serves as a robust platform for accelerating the prediction of oxidation potential (*E*_ox_). Using the PBE0 hybrid functional and cc-pVDZ basis set, we establish a strong near-linear correlation between *E*_HOMO_ and experimental *E*_ox_ values, achieving an exceptional correlation coefficient (*R* ^2^) of 0.977 and a low root-mean-square error (RMSE) of 0.064. The correlation highlights the accuracy of OxPot as a machine learning (ML)-ready resource for *E*_ox_ prediction. To further facilitate future development of ML models, we extensively tested various algorithms and conducted a thorough feature importance analysis. This analysis offers valuable insights into the key molecular descriptors that influence *E*_ox_ predictions, thereby enhancing model interpretability and guiding the design of more effective predictive models. Furthermore, the computational efficiency of the methodology ensures rapid predictions of *E*_ox_ for additional chemically similar molecules, thereby increasing its applicability for large-scale molecular screening and broader applications in chemical research.

## Graphical abstract

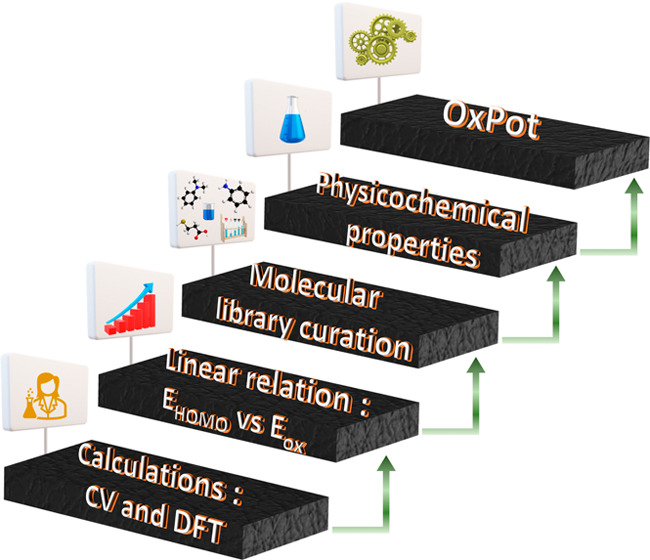

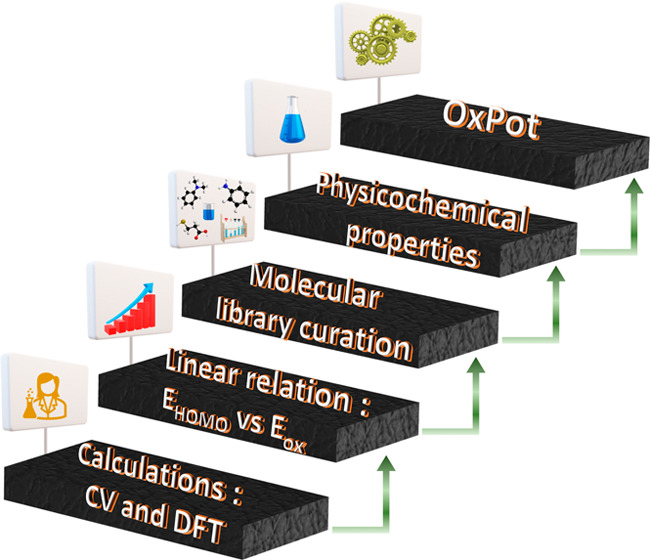

## Introduction

The global surge in energy demand has amplified the search for advanced materials capable of driving effective and sustainable energy-related chemical transformation. Redox-active organic molecules have emerged as promising candidates due to their capacity to participate in electron-transfer reactions crucial to applications such as organic photovoltaics, catalysis, energy storage, and electrochemical water treatment.^1,2^ A critical parameter governing the reactivity of these molecules in redox reactions is the *E*_ox_, which quantitatively measures a molecule’s ability to donate electrons in a specific environment. *E*_ox_ provides insights into molecular stability, electron-donating or withdrawing tendencies, and applicability across diverse fields. For instance, in photovoltaics, *E*_ox_ influences the alignment of energy levels between donor and acceptor species, which is vital for effective charge transfer. In catalysis, it governs the molecular participation in redox reactions, while in electrochemical cells, *E*_ox_ determines the ease of oxidation of the electrolyte additive, impacting system efficiency and voltage stability.^3,4^

Traditional experimental methods for estimating the *E*_ox_ include cyclic voltammetry (CV), differential pulse voltammetry, square wave voltammetry, and pulse radiolysis.^5^ These are considered the gold standard for measuring *E*_ox_. Although highly accurate, these methods are technically quite complex, require specialized instrumentation, and are time-consuming as they can only predict the *E*_ox_ of one molecule at a time, significantly hindering their utilization in large chemical spaces.^6^ Computational methods, such as Hartree–Fock and DFT, provide a robust alternative for predicting *E*_ox_.^7^ Despite their reliability, these computational methods are limited by their high computational cost, which scales poorly with molecular size and complexity. Such drawbacks make traditional and computational methods impractical for high-throughput screening across large chemical libraries, posing a substantial challenge in discovering redox-active candidates optimized for energy applications.

To bridge this gap, machine learning (ML) offers a powerful tool for predicting molecular properties, including *E*_ox_, with the potential for high-throughput capability and rapid scalability. ML excels at identifying patterns within large data sets and can generate fast,^8^ accurate predictions at a fraction of the computational cost of DFT. Furthermore, ML models trained on diverse data sets, such as OxPot, can generalize predictions across chemically diverse molecules, supporting scalable predictions for high-throughput applications. By combining the speed and scalability of ML with the detailed accuracy of DFT, we can mitigate the limitations of traditional quantum methods while ensuring precise *E*_ox_ predictions essential for advancing applications in catalysis, energy storage, and beyond.

In this work, we introduce OxPot, a comprehensive, open-access data set comprising oxidation potential data for 15,238 organic molecules with high potential for energy applications. The majority of the previous databases predominantly focus on organic solvents,^9–11^ however, OxPot focuses on *E*_ox_ predictions in the aqueous phase, thereby providing a valuable resource for applications where water stability is essential, such as in water-based redox flow batteries and electrochemical catalysis. Compared to organic solvents, the solubility, hydrogen bonding, and stability of molecules can vary significantly in aqueous regions, and these factors directly affect the *E*_ox_ of a molecule. For instance, in water-based flow batteries, the stability of the aqueous phase significantly affects the efficacy and lifetime, underscoring the importance of accurate aqueous-phase predictions.

Furthermore, we have included a comprehensive study in which various classical and advanced ML models were trained on the OxPot data set. The aptness of OxPot as a training data set has been demonstrated, providing a foundation for future research. This data set offers the opportunity to investigate other critical molecular properties, further advancing applications in energy-related fields.

## Methods

Experimental details and computational settings used in the analysis are summarized in the Supporting Information Sections S1 and S2. The OxPot data set was sourced from PubChem, a well-established online database platform, and includes a wide range of relevant physicochemical properties of molecules that can be identified using their unique compound identifier (CID).^12^ Key molecular characteristics were either extracted from established databases or predicted using open-source tools. For example, SMILES, InChI, and molecular weight were extracted from PubChem. RDKit^13^ was used to predict the topological polar surface area, number of hydrogen bond donors, number of hydrogen bond acceptors, aromatic rings, and fractional Csp3. An open-source ML model, AqSolPred,^14^ trained on a refined version of AqSolDB,^15^ was utilized for solubility predictions.

According to Koopmans’ theorem, the vertical ionization energy is approximated by the energy of the occupied molecular orbitals.^16^ Although Koopmans’ theorem is strictly valid within the restricted Hartree–Fock framework, a similar concept also applies within the DFT formalism.^17^ This concept forms the basis for the application of energetic descriptors, such as *E*_HOMO_ or *E*_LUMO,_ in predicting redox potential, and the linear relationship between them has been widely explored in literature studies,^18–21^ facilitating a more straightforward method for estimating the redox potentials of unknown molecules. Therefore, by calculating the *E*_HOMO_ values for each molecule using DFT and employing an empirically derived linear correlation with experimental *E*_ox_, we provide accurate *E*_ox_ predictions that can be used for training ML models. This comprehensive data set is further validated against literature-reported values, demonstrating strong agreement and establishing OxPot as a robust resource for training ML algorithms that can aid in *E*_ox_ prediction.

The OxPot data set provides detailed information on the *E*_ox_ and various chemical properties of molecules. The data is conveniently stored in a CSV file, making it easily accessible for researchers. Each row in the CSV file represents a single molecule, while the 16 columns correspond to either a chemical or quantum property or an identifier. Specifically, the data set includes three molecular identifiers, InChI, SMILES, and CID; two quantum properties, *E*_HOMO_ and *E*_ox_; and 11 chemical properties. In addition to the CSV file, we also provide a compressed tar.gz file containing XYZ files with the 3D conformations of the molecules, which were used as input for DFT calculations. These 3D conformations, representing the atomic coordinates of each molecule, were generated using the ETKDG method^22^ that provided an in-depth exploration of the molecular structures.

## Results

### OxPot Data Set

The data in OxPot is created using first-principles and experimental CV studies, which were adequately parametrized. The data produced in OxPot is fully reproducible to numerical precision, provided the specified parameters and protocols are strictly adhered to. Therefore, any uncertainties are attributed to the applied modeling parameters and the underlying data quality. We used CV and DFT studies to determine the characteristic quantum properties, *E*_ox_, and *E*_HOMO_ of the commonly available yet chemically diverse organic molecules. In CV, most of the molecules underwent single-electron transfer, and we used the maximum anodic peak potential (E_pa_) to report the potential. In OxPot, we primarily considered that molecules undergo single-electron transfer, with the potential for multielectron transfer under specific conditions.

The steps involved in constructing the OxPot data set are outlined in Scheme 1. These include *E*_ox_ measurements, physics-based calculations on molecules, ML predictions of the physicochemical properties of compounds, and the creation of the OxPot model. The systematic development of the virtual library starts with measuring *E*_ox_ using CV and calculating *E*_HOMO_ via DFT. This process establishes a linear relationship between these two factors, which is then used to predict the *E*_ox_ of molecules curated from PubChem using a specific set of rules. The physicochemical properties of the molecules are derived either from open-source tools or using ML models. Finally, the results are extracted, processed, and organized into a relational database by parsing the output files from the first-principles calculations and ML models.

**Scheme 1:**
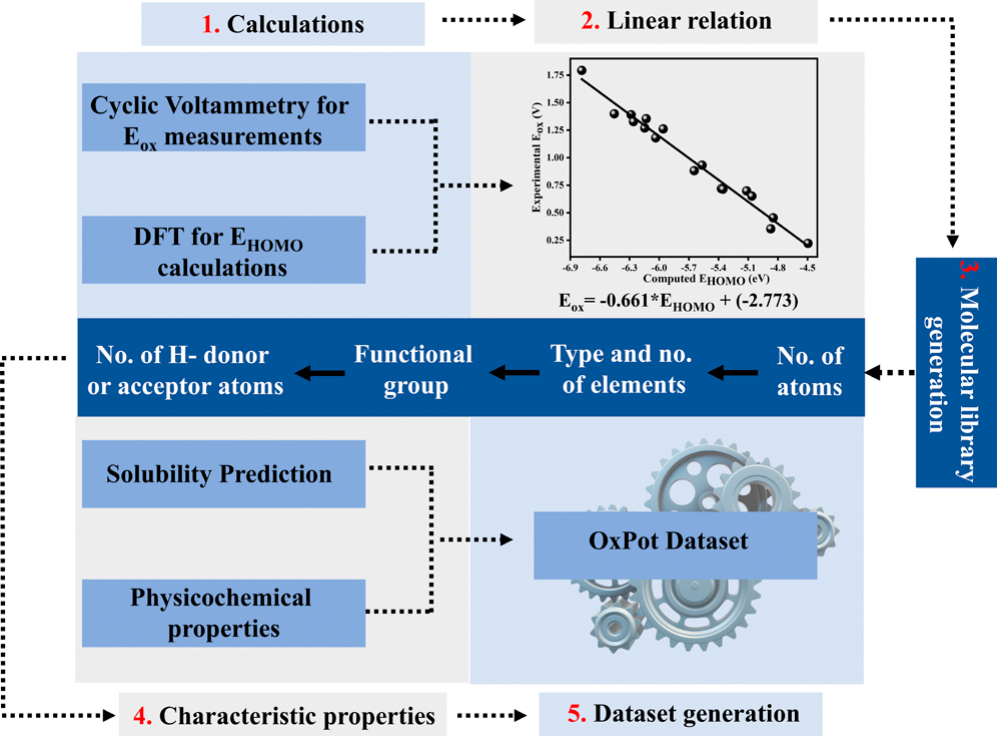
Schematic Overview of the Steps Involved in the Development of OxPot

The linear correlation between computed *E*_HOMO_ and electrochemical *E*_ox_ ^16,23^ resulted in an *R* ^2^ value of 0.977, along with a slope of −0.661 and an intercept of −2.773, demonstrating a strong linear dependence of *E*_ox_ on *E*_HOMO_ (Figure 1). High *R* ^2^ value proposes that the linear model delivers adequate accuracy for predicting *E*_ox_ from *E*_HOMO_. The negative slope highlights the chemical trend that an increase in *E*_HOMO_ corresponds to a decrease in *E*_ox_, indicating enhanced ease of oxidation for molecules with higher *E*_HOMO_ levels. This trend aligns well with the fundamental principles of redox chemistry, where oxidation becomes thermodynamically favorable for molecules with higher *E*_HOMO_.

**Figure 1:**
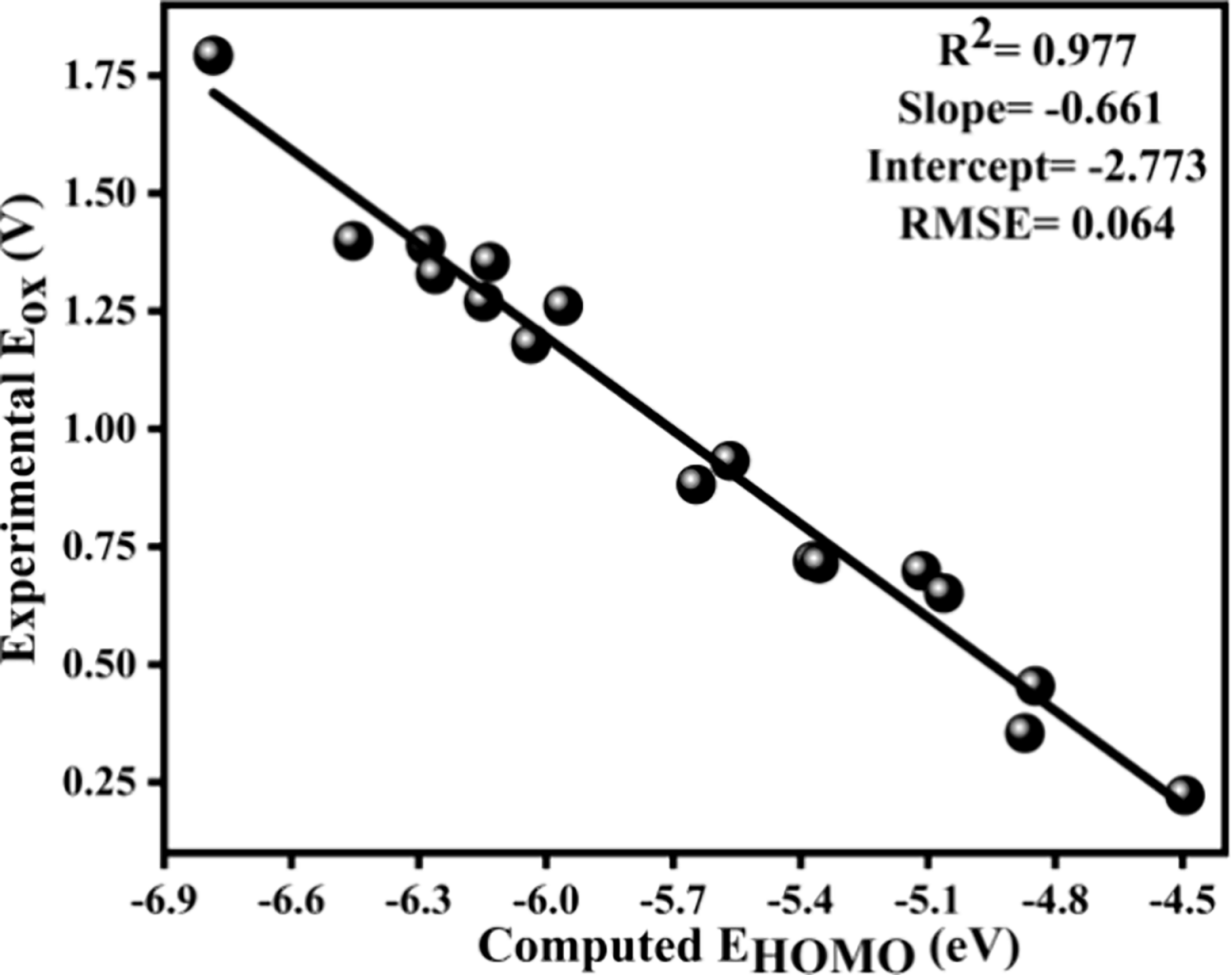
Linear relationship between computationally predicted *E*_HOMO_ and experimentally measured *E*_ox_.

Figure 2 shows the excellent alignment between the experimental and predicted *E*_ox_ values. The experimentally measured and predicted *E*_ox_ values for all tested molecules are listed in Table S1, along with the calculated differences. The accuracy of the predicted *E*_ox_ values, as illustrated by the small deviations, highlights the robustness of the linear regression approach and the validity of using *E*_HOMO_ as a descriptor for redox potential predictions. For DFT calculations, the PBE0 hybrid functional^24^ was selected as it incorporates 25% exact exchange from Hartree–Fock theory and the generalized gradient approximation (GGA) for improved accuracy in predicting molecular properties. Combined with the correlation-consistent polarized valence double-ζ (cc-pVDZ) basis set, it offers a good balance between computational efficiency and accuracy.

**Figure 2:**
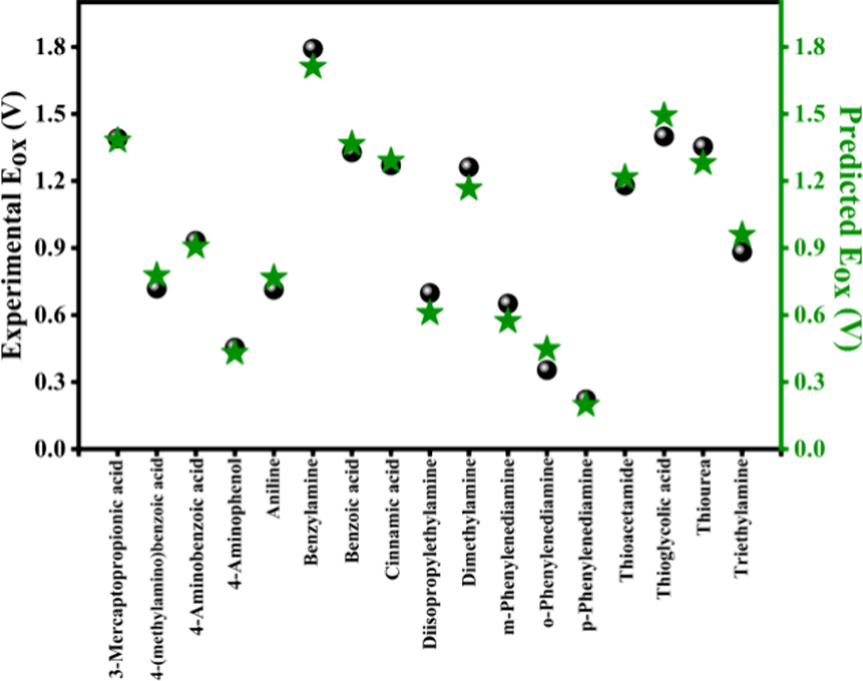
Comparison of experimental (spheres) and predicted (stars) *E*_ox_.

Various functionals and basis sets were tested for the DFT calculations of *E*_HOMO_ for the set of molecules whose *E*_ox_ were measured using CV. The selection criteria for functionals and basis sets included prior studies that demonstrated their effectiveness in predicting electronic properties relevant to *E*_ox_. Among the combinations tested, the hybrid functional PBE0 paired with the cc-pVDZ basis set and the Restricted Kohn–Sham approach showed the best performance, achieving an *R* ^2^ value of 0.977 and RMSE of 0.064 (Figure 3). This accuracy highlights the suitability of these parameters for predicting *E*_HOMO_, which can be used to estimate the *E*_ox_ of molecules. A more detailed performance assessment of the different functional and basis set combinations is provided in Table S2.

**Figure 3:**
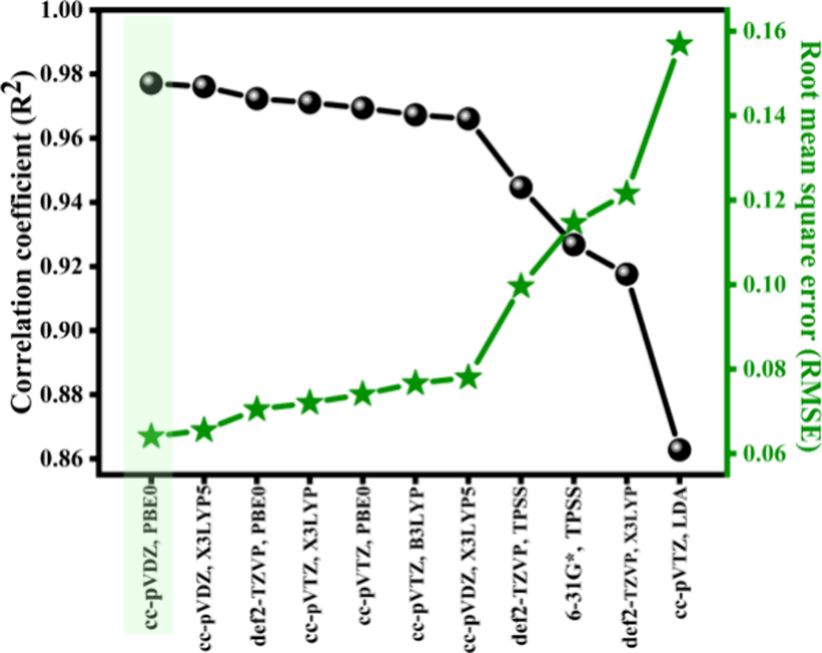
Performance comparison of a few functionals considered in the current work (stars for RMSE and spheres for *R* ^2^).

A set of rules was applied to filter the molecules from PubChem, developing an enhanced data set with high prediction precision. Detailed criteria for this filtering process are summarized in the Supporting Information Section S3. Figure 4a,b illustrates the distribution of molecules based on the functional group and their corresponding distribution range of predicted *E*_ox_ value in OxPot. Most molecules in OxPot contain either an amine or carboxyl group, with a smaller subset featuring combinations of functional groups such as amine and alcohol or amine and carboxyl or amine/alcohol and thiol. The predicted *E*_ox_ values range from −0.6 to 2.7 V, with most molecules exhibiting values between 0.4 and 1.4 V (Figure S1). The vast majority of the *E*_ox_ values follow well-established chemical trends. As expected, the data in Figure 4c,d show that molecules with electron-donating functional groups, such as amines, have lower *E*_ox_ values than those with electron-withdrawing groups, like carboxyl groups. For example, the *E*_ox_ of aniline (0.714 V vs Ag/AgCl (Sat. KCl)) is significantly lower than that of cinnamic acid (1.270 V vs Ag/AgCl (Sat. KCl)). This can be attributed to the electron-donating nature of the amine group, which facilitates oxidation more easily compared to the carboxyl group, which withdraws electron density, making the molecule more difficult to oxidize. Additionally, the *E*_ox_ decreases with the increase in the number of electron-donating groups in the molecule. For instance, triethylamine (0.882 V vs Ag/AgCl (Sat. KCl)) has a lower *E*_ox_ than dimethylamine (1.261 V vs Ag/AgCl (Sat. KCl)), likely due to the increased electron-donating effect of the additional alkyl groups, which help stabilize the radical cation.

**Figure 4:**
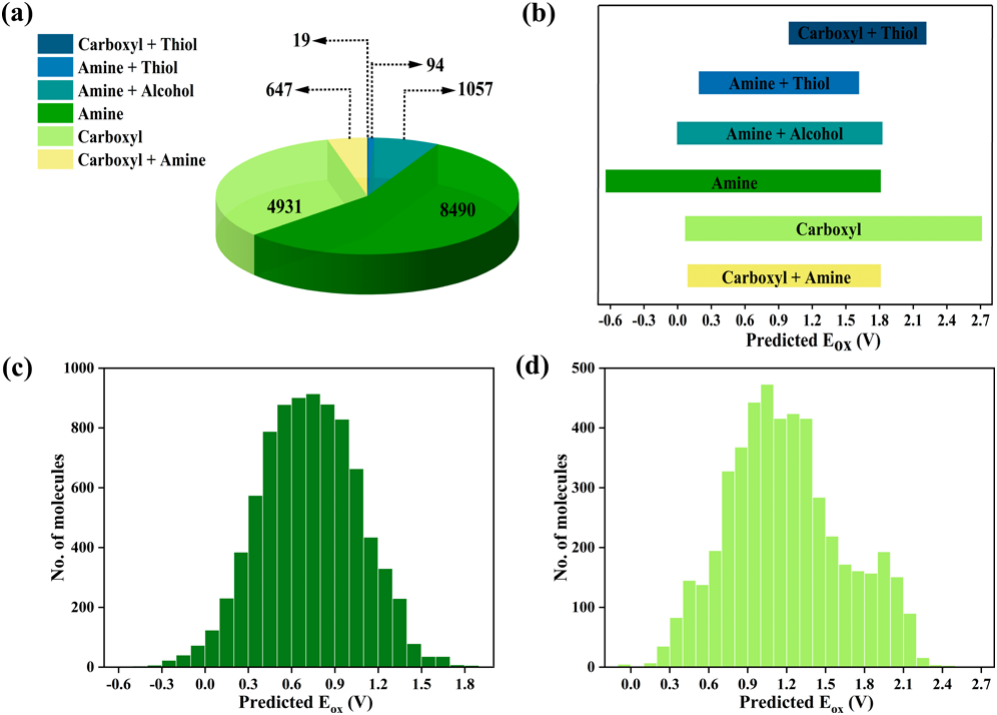
Distribution of molecules in the OxPot data set based on (a) the type of functional groups present, (b) the predicted *E*_ox_ range for different functional group molecules, (c) the predicted *E*_ox_ values for amine-containing molecules, and (d) the predicted *E*_ox_ values for carboxyl-containing molecules.

To validate the accuracy of OxPot, we compared the predicted *E*_ox_ values with those of several molecules reported in the literature.^25–30^ It is essential to note that the molecules tested from the literature were not used to determine the intercept and slope of the linear relationship between *E*_ox_ and *E*_HOMO_, thereby ensuring independent validation. The experimental value can vary depending on the type of reference electrode used; therefore, these values were corrected. Both literature and computed potentials were referenced to Ag/AgCl (sat. KCl), which was consistent with the conditions used in our predictions. Figure 5 displays a plot of the predicted *E*_ox_ values against the literature values, showing a robust and near-linear correlation with an *R* ^2^ value of 0.963 and a mean absolute error (MAE) of 0.069, indicating that the OxPot data set effectively captures the relationship between *E*_HOMO_ and *E*_ox_, even for molecules with diverse chemical structures and experimental conditions which reinforces the reliability of our approach. The minor deviations likely arise from differences in experimental setups or solvent effects that are not explicitly captured in the computational model. In addition, we also incorporated molecules whose *E*_ox_ values were measured in organic solvents, such as acetonitrile. Despite the focus of the OxPot data set on aqueous systems, a high *R* ^2^ value was observed even with the addition of these molecules, signifying that the predictive model generalizes well with the organic solvents too. This generalization is likely due to the implicit solvent model, COSMO, used during the DFT calculations, which enabled a versatile approximation of solvation effects across different environments. The results validate the model’s accuracy and demonstrate its tendency to generalize across diverse chemical environments. A complete description of solubility estimation is discussed in the Supporting Information Section S4.

**Figure 5:**
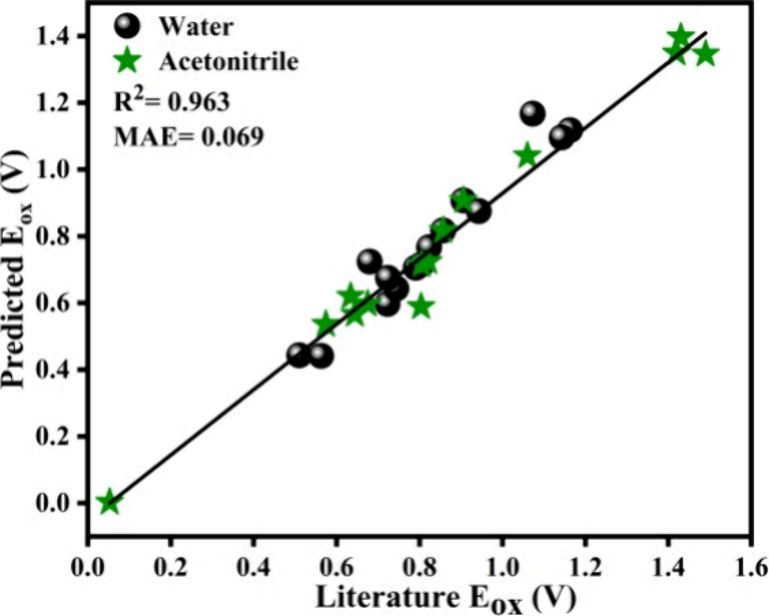
Predicted vs literature *E*_ox_ (black spheres for water and green stars for acetonitrile).

To establish the suitability of the OxPot data set for training ML models, we first evaluated whether the data set contains meaningful patterns rather than noise. Specifically, we assessed whether various ML algorithms could successfully converge during training and achieve strong predictive performance. Full details of the model’s setup are provided in Section S5 of the Supporting Information.

We trained several classical ML algorithms, including Random Forest, Support Vector Machine (SVM) with both linear and radial function kernels, and a Multilayer Perceptron (MLP), using Morgan fingerprints^31^ as input features. These fingerprints are widely used in molecular prediction tasks, providing a robust, chemically informed representation of molecular structure. In addition to classical approaches, we also evaluated Message Passing Neural Networks, a class of Graph Neural Networks (GNNs) specifically designed to process graph-structured data such as molecular graphs. GNNs represent the current state-of-the-art in molecular property prediction, mainly when applied to relatively large data sets such as OxPot.

We used two different architectures in our experiments: a Graph Convolutional Network (GCN),^32^ a widely adopted and well-established model, and Graph Isomorphism Network (GIN),^33^ a more recent and expressive architecture that has demonstrated superior performance across many molecular benchmarks. In this setting, each molecule is represented as a graph, where atoms correspond to nodes and chemical bonds correspond to edges. GNNs operate by iteratively updating the embeddings of each node based on the features of its neighboring nodes and the connectivity of the graph. At each layer, information is aggregated from adjacent atoms and combined to form richer, context-aware node embeddings. After several message-passing steps, these embeddings capture both local atomic patterns and global molecular structure. A pooling layer then combines the node embeddings into a single graph-level representation, which is used to predict molecular properties. A more detailed mathematical formulation is available in Section S6 of the Supporting Information.

The above-mentioned ML models were assessed based on two different evaluation criteria: MAE and RMSE. The smaller the values of MAE or RMSE are, the higher the performance of the model. The results, summarized in Table 1, show that all models except Linear SVR achieved a MAE of approximately 0.12 or lower with low standard deviation, indicating that the models could reliably converge and extract relevant patterns from the data. These results also suggest that a simple linear model such as Linear SVM is insufficient to capture the relationship between molecular features and E_ox_. Among all models, GIN achieved the best performance, with an MAE of 0.074 and RMSE of 0.116, outperforming all other baselines significantly. These findings support the conclusion that the OxPot data set is well-suited for predictive modeling of *E*_ox_.

**Table 1:** MAE and RMSE Values of Tested Algorithms

**method**	**MAE**	**RMSE**
random forest	0.107 ± 0.002	0.189 ± 0.03
SVM	0.115 ± 0.004	0.185 ± 0.035
linear SVM	0.163 ± 0.006	0.241 ± 0.031
MLP	0.106 ± 0.005	0.172 ± 0.031
GCN	0.09 ± 0.002	0.14 ± 0.014
GIN	0.074 ± 0.003	0.116 ± 0.008

The atom-level feature descriptors used in both GIN and GCN models include a range of properties such as atomic number, chirality, number of directly bonded neighbors (NDBN), number of bonded hydrogens (NBH), formal charge, number of radical electrons (NRE), hybridization, aromatic, ring, TPSA, fraction csp3, LogP, pH, oxidation state, and molecular mass, explanation for each feature can be found in the Supporting Information Section S5.

To further interpret the model’s behavior and assess which features contributed most to its predictions, we applied GNNExplainer^34^ to the trained GIN model, which demonstrated the best performance. GNNExplainer is a specialized explanation method for GNNs, analogous to SHAP (SHapley Additive exPlanations)^35^ that identifies the most influential input features in the model’s decision-making process. As shown in Figure 6, the most critical atomic features identified include oxidation state, NBH, and NDBN, properties that are known to correlate with or influence strongly the *E*_ox_ of the molecule. These features align perfectly with the known theoretical frameworks and also deliver new insights into how local atomic environments and electronic structure impact the *E*_ox_, such as the oxidation state of an atom, which reflects its electron-donating or -withdrawing nature, significantly influences the molecule’s overall reactivity and, hence, the *E*_ox_ value.

**Figure 6:**
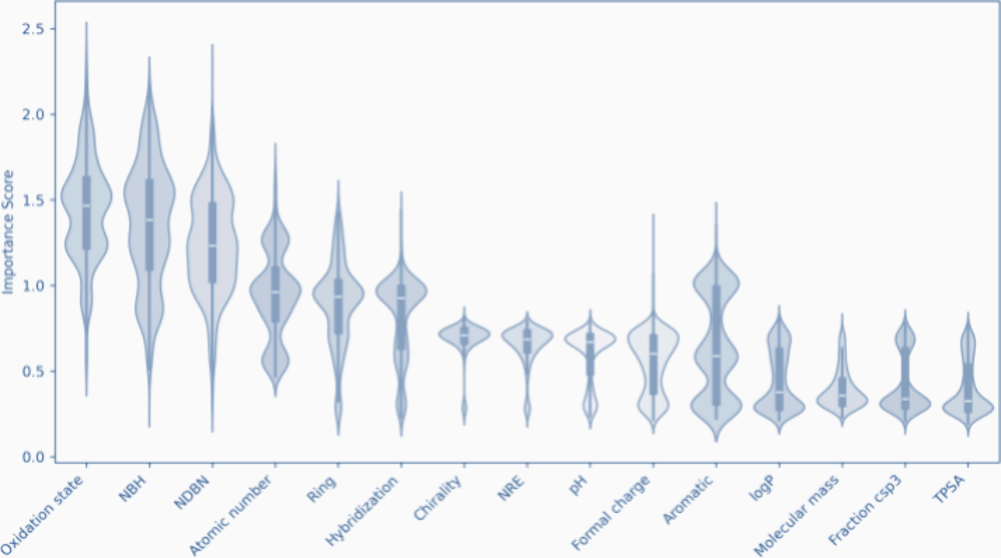
Violin plot showing the distribution of importance scores for each atom-level feature across the data set, as computed by GNNExplainer for the GIN model.

The comparison of *E*_ox_ values predicted by the ML-GIN model with those from OxPot demonstrates OxPot’s ability to provide an efficient data set with meaningful and accurate values that could be employed to train ML models. A pool of molecules with the comparison values is given in Figure 7. Furthermore, the *E*_ox_ values of molecules with different functional groups aligns perfectly with the established chemical understanding, i.e. molecules with electron-donating groups (e.g., amines) tend to have lower E_ox_ values as compared to with electron-withdrawing groups (carboxyl groups). A similar trend was also discussed in Figure 4. These findings underscore the potential of training an ML model with OxPot to enhance the prediction accuracy of *E*_ox_ and further our understanding of molecular redox behavior.

**Figure 7:**
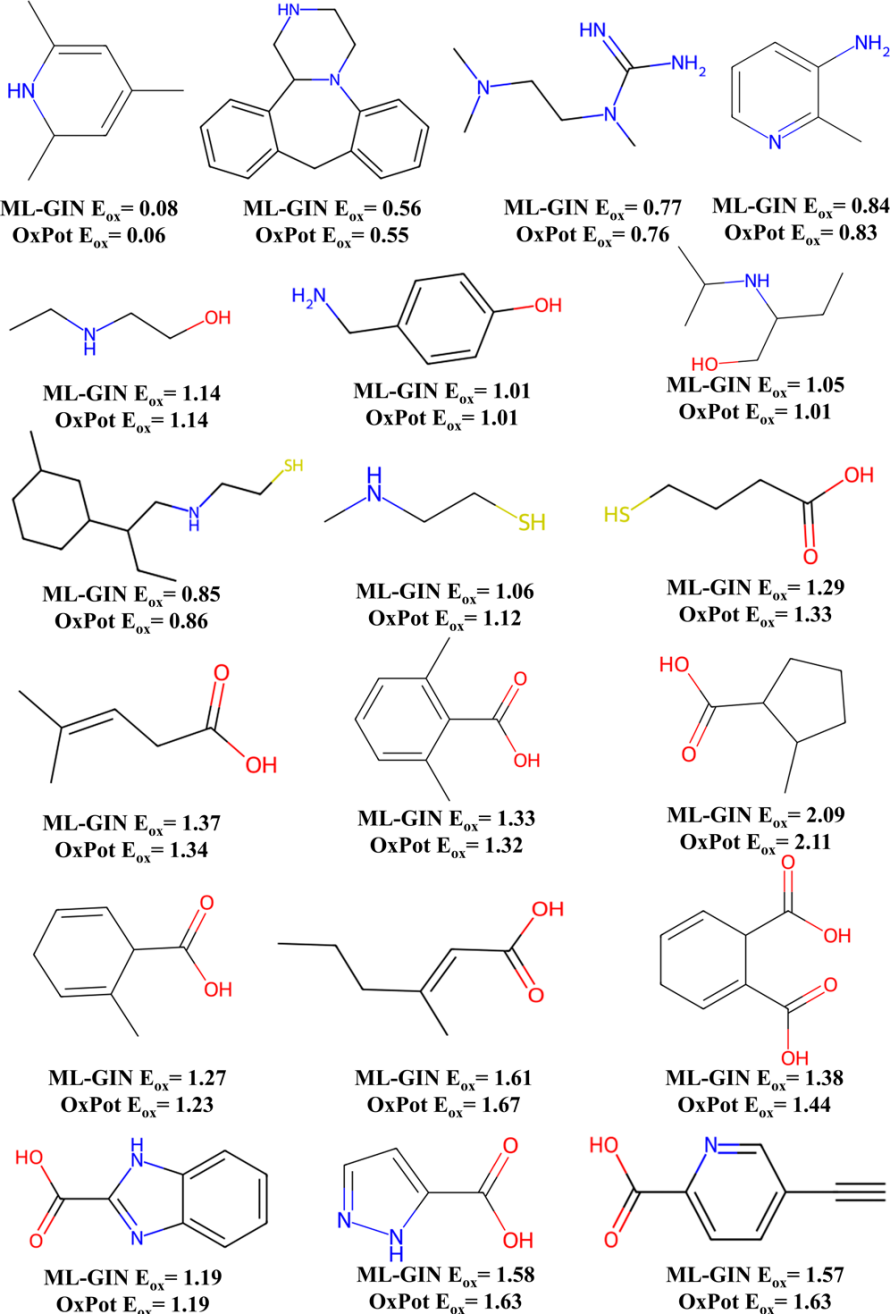
Comparison of predicted *E*_*ox*_ from OxPot and by ML-GIN.

## Conclusions

In summary, a machine learning-ready data set, OxPot, was proposed for predicting the oxidation potential of organic molecules, which is fully reproducible, transparent, and readily accessible. Special consideration was given while curating molecules from PubChem to enlist structurally and chemically similar molecules to the experimental molecules. While the experimental measurements were performed exclusively in aqueous solution, the predicted results showed their accuracy even in organic solvents, probably due to the robustness of the implicit solvation model employed during DFT calculations. The OxPot data set stands out due to its combination of high prediction accuracy and the incorporation of experimental measurements of *E*_ox_. Furthermore, a preliminary study of training machine learning models using the OxPot data set confirmed its suitability and alignment with the hypothesis of the proposed work. Thus, we envision that the current study will empower researchers to develop predictive models that have the potential to screen extensive molecular libraries, thereby facilitating the discovery of new materials optimized for diverse energy-related applications.

## Supplementary Material





## Data Availability

All DFT calculations were conducted using the open-source Python library PySCF,^36^ distributed under the Apache-2.0 license. The PySCF library can be accessed through its GitHub repository (https://github.com/pyscf/pyscf). The 3D conformations of the molecules used in the DFT calculations were generated using RDKit, an open-source cheminformatics library available under the BSD license. The RDKit library can be found on GitHub (https://github.com/rdkit/rdkit). Solubility estimates were obtained using AqSolPred, an openly accessible tool. The code used to create the Open Oxidation Data set is available in GitHub repository (https://github.com/nkami/oxpot/tree/main) also under the Apache-2.0 license.

## Notes

Additional details on the experimental procedure for *E*_ox_ measurement, DFT calculations for *E*_HOMO_, steps for molecular library generation, solubility prediction, figures depicting the *E*_ox_ distribution of molecules in OxPot, tables comparing experimental *E*_ox_ values with predicted values, and linear fitting data for different combinations of DFT functionals (PDF)
OxPot data set (XLSX)
3D conformations of the molecules (ZIP)
