# Supplementary material for: Predicting Oxidation Potentials with DFT-Driven Machine Learning
Source: J Chem Inf Model. 2025 May 28;65(11):5345–51. doi: 10.1021/acs.jcim.5c00159 (PMC12225624; doi:10.1021/acs.jcim.5c00159)
Supplement: Supplementary file 1 [file ci5c00159_si_001.pdf]

Supporting Information

Predicting Oxidation Potentials with DFT-Driven Machine Learning

Shweta Sharma[1§], Natan Kaminsky[2§], Kira Radinsky[2*], and Lilac Amirav[1*]

[1]Schulich Faculty of Chemistry, and [2]Taub Faculty of Computer Science, *Technion − Israel Institute of Technology, Haifa 32000, Israel*

[§] Both authors contributed equally

S1. Oxidation Potential Measurements by CV

We measured the E_{ox} of commonly available yet chemically diverse organic molecules using CV. These molecules were selected based on the plausibility of their potential as redox-active molecules. CV is a powerful electrochemical technique employed for probing the redox behavior of molecular species. In the present study, experiments were performed in a 20 mL glass cell containing 1 M potassium chloride in water and 0.01 M organic molecules of interest. The working electrode was a glassy carbon rod with sides encased in Teflon, polished/cleaned on a microfibre cloth using aqueous alumina and diamond suspension, and the counter electrode was a Platinum wire. The reference electrode was Ag/AgCl (saturated KCl) (+0.197 V_{NHE}), and if not otherwise specified, potentials are reported against Ag/AgCl (sat. KCl) throughout the study. Prior to measurement, the system was purged with Argon to remove dissolved oxygen. A background CV scan was recorded before each experiment to eliminate background impurities. Each scan was analyzed in terms of the maximum anodic peak potentials.

S2. Estimation of Oxidation Potential by DFT

DFT does not directly calculate the E_{ox} of molecules; however, we can estimate this property by leveraging molecular descriptors that exhibit a strong correlation with E_{ox}, such as the energy of the highest occupied molecular orbital, E_{HOMO}, which can be obtained through computational DFT calculations. According to the Marcus theory of electron transfer and several simplifying approximations from prior works,[1, 2] it is reasonable to assume a linear relationship between a neutral molecule's E_{ox} and its E_{HOMO} energy. This relationship can be expressed as:

$$E_{OX/RED} = a + b \cdot E_{HOMO/LUMO}$$

Where $E_{OX/RED}$ denotes the experimental oxidation or reduction potentials; $E_{HOMO/LUMO}$ represents the calculated HOMO and LUMO energies; and a and b are constants. The constants a and b were determined through calibration against experimental E_{ox} data of a reference set of molecules, which have been measured by CV. This approach is well-established and has been adopted in several previous studies[3-6]. Once a correlation between E_{HOMO} and E_{ox} is established , it can be applied to predict the E_{ox} value of chemically similar organic molecules.

To generate the initial molecular conformations for the DFT calculations, we utilized the Experimental-Torsion Knowledge Distance Geometry (ETKDG) method, implemented through

RDKit.[7] We investigated various combinations of DFT functionals, basis sets, and solvation radii to identify the optimal parameters for improving the predictive accuracy of OxPot. A total of four functionals, namely PBE0, X3LYP5, X3LYP, and B3LYP, and five basis sets, namely cc-pVDZ, 6-311G**, 6-31G*, cc-pVTZ, and def2-TZVP, were used for the computational study. We employed the implicit solvation model COSMO (ddCOSMO),[8] which effectively captures solvation effects using a domain decomposition method to model the aqueous environment in which many oxidation reactions occur. The calculations were performed using the open-source Python library PySCF,[9] ensuring precise modelling of solvated molecules.

S3. Molecule Library Generation

The OxPot data set consists of 15,238 molecules sourced from PubChem, ensuring that the compounds are accessible and feasible as redox-active molecules. To enhance the accuracy of the predicted E_{ox}, the data set was curated based on the experimental CV studies and computational limitations. For example, the total number of atoms in each molecule was restricted to a maximum of 40 as the DFT calculations scale poorly with molecular size, leading to significantly increased computation time for larger molecules. Moreover, OxPot was limited to molecules containing five elements: Carbon (C), Hydrogen (H), Oxygen (O), Nitrogen (N), and Sulphur (S). This selection provides diverse organic molecules while keeping computational analysis manageable. The functional groups present in molecules were amines (primary, secondary, and tertiary), alcohol (-OH), carboxyl (-COOH), and thiol (-SH). These functional groups were chosen considering the available chemical knowledge of their ability to tune the E_{ox} and aqueous solubility of the molecules. Furthermore, the number of hydrogen donor or acceptor atoms in each molecule was limited to a maximum of two. This restriction is necessary because the relationship between a molecule's E_{ox} and H-donor or acceptor ability is fundamentally rooted to its electronic structure and the thermodynamics of redox reactions. A higher number of hydrogen donor or acceptor atoms increases interactions with water molecules through hydrogen bonding, which can significantly reduce DFT precision.

S4. Estimation of Solubility

Accurately estimating the solubility of a molecule is a complex challenge, yet it is a critical factor when integrating molecules into real-world applications. Solubility plays a crucial role in determining the feasibility of a molecule in such systems, where its ability to dissolve efficiently in solvents like water is essential for optimal performance. To address this challenge, we rely on the ML model, AqSolPred,[10] which has been used previously to estimate the solubility of various organic molecules in water.[11] AqSolPred is an ensemble of ML models. It was trained on a refined version of AqSolDB,[12] a large dataset that compiles reference data on aqueous solubility. The input to AqSolPred consists of SMILES (Simplified Molecular Input Line Entry System) representations of the target molecules, a standard format in cheminformatics. The model converts these SMILES strings into molecular fingerprints, which are rich vector representations capturing the chemical properties and structural features of the compounds. These fingerprints are then used as inputs to the model's predictive algorithms. AqSolPred's output is a solubility score in LogS units (log of mol/L), a logarithmic solubility measure commonly used in ML tasks related to this property. We convert the model output into a more intuitive format—milligrams per liter (mg/L) to enhance interpretability. This conversion allows for a more straightforward evaluation of whether a chemical compound meets the solubility requirements for its intended application, where solubility thresholds can be critical for effective performance. According to the original AqSolPred study, the model achieved a MAE of 0.348 LogS on the Huuskonen dataset,[13] a widely recognized benchmark for evaluating the performance of solubility prediction models. AqSolPred outperformed all other baseline models and is regarded as a state-of-the-art method for predicting solubility in water for chemical compounds.

S5. Machine Learning Models: Experimental Settings

All experiments were conducted five times, each with a different random seed to ensure robustness and reproducibility of results. For each run, the dataset was split into training (80%), validation (10%), and test (10%) sets.

Hyperparameter tuning was performed independently for each method using the validation set to identify the best-performing configuration. Once the optimal hyperparameters were determined,

the corresponding model was retrained on the training set and evaluated on the test set to assess generalization performance.

For graph neural network models, the validation set was also used to apply early stopping during training, preventing overfitting and ensuring better model generalization. For the classical machine learning algorithms, we used 2048-bit Morgan fingerprints with a radius of 2 as molecular descriptors. These fingerprints capture circular substructures around each atom, encoding molecular topology in a fixed-length binary vector.

In contrast, graph neural network models received molecules in graph form, where atoms were treated as nodes and bonds as edges, allowing the models to learn directly from molecular structure. Each atom was described using a one-hot encoded feature vector composed of the following properties:

- **Atomic Number** – Identifies the element type (e.g., carbon, oxygen, nitrogen) of the atom.
- **Chirality** – Indicates the stereochemical configuration (e.g., R/S) of the atom, relevant for enantiomeric distinctions.
- **Oxidation State** – Represents the formal oxidation number, computed using Pauling electronegativity scaling to estimate electron distribution.
- **Number of Directly-Bonded Neighbors (NDBN)** – Counts the immediate bonded atoms, reflecting local atomic connectivity.
- **Formal Charge** – The net electric charge assigned to the atom, assuming standard bonding.
- **Number of Bonded Hydrogens (NBH)**– The number of hydrogen atoms directly attached to the atom.
- **Number of Radical Electrons (NRE)** – Indicates unpaired electrons, which are relevant for reactivity.
- **Hybridization** – Specifies the atom’s orbital hybridization state (e.g., sp, sp^2, sp^3), affecting molecular geometry.
- **Aromatic** – A binary flag indicating whether the atom is part of an aromatic system.
- **Ring** – A binary feature showing if the atom is part of any ring structure in the molecule.
- **Topological Polar Surface Area (TPSA)** – A measure of the atom’s contribution to the molecule's polar surface area, related to solubility and permeability.

- **Fraction of sp³ Carbons (Fraction csp3)** – Indicates the degree of carbon saturation in the molecule, a proxy for 3D character and drug-likeness.
- **LogP** – The predicted octanol-water partition coefficient, representing molecular hydrophobicity.
- **pH** – The pH at which the molecule is likely neutral was estimated based on the presence of known acidic and basic functional groups. Using SMARTS patterns, we identified the strongest acidic and basic pKa values and computed the neutral pH as their average. If only one type was present, the pH was set to one unit below (acid) or above (base) the corresponding pKa. If no groups were detected, a default value of 7 was used.
- **Molecular Mass** – The total molecular weight of the compound, included for completeness and global context.

Features that describe the molecule as a whole (such as TPSA, logP, pH, molecular mass, and fraction csp3) were repeated identically for all atoms in the input graph, ensuring that each atom had access to global molecular context.

S6. Graph Neural Networks Formulation

Graph Neural Networks are a class of neural networks designed to operate on graph-structured data. Formally, consider a graph $G = (V, E)$ where V is a set of nodes and $E \subseteq V \times V$ is a set of edges. Each node $v \in V$ is associated with a feature vector $x_v \in \mathbb{R}^d$. The network iteratively updates the representation of each node using information from its neighbours. In this process, the number of layers in the network corresponds to the number of iterations. Let $h_v^{(k)}$ denote the hidden representation of node v after k iterations (or layers). Initially, we have:

$$h_v^{(0)} = x_v$$

During each iteration the nodes are updated by messages from their neighbours and their current representation:

$$m_v^{(k)} = \boldsymbol{\Psi}\left(\left\{\varphi_\theta\left(h_u^{(k-1)}\right) : u \in N(v)\right\}\right)$$

$$h_v^{(k)} = \psi_\theta\left(h_v^{(k-1)}, m_v^{(k)}\right)$$

Where $\psi_\theta, \varphi_\theta$ are neural networks, $\boldsymbol{\Psi}$ is a permutation invariant function (e.g., sum, mean, or max) that combines messages from all neighbours $N(v)$ of node v**.** At the final iteration, the node representations are aggregated using a permutation-invariant function to produce a single vector representing the entire graph, which can then be used for tasks such as graph classification or regression.

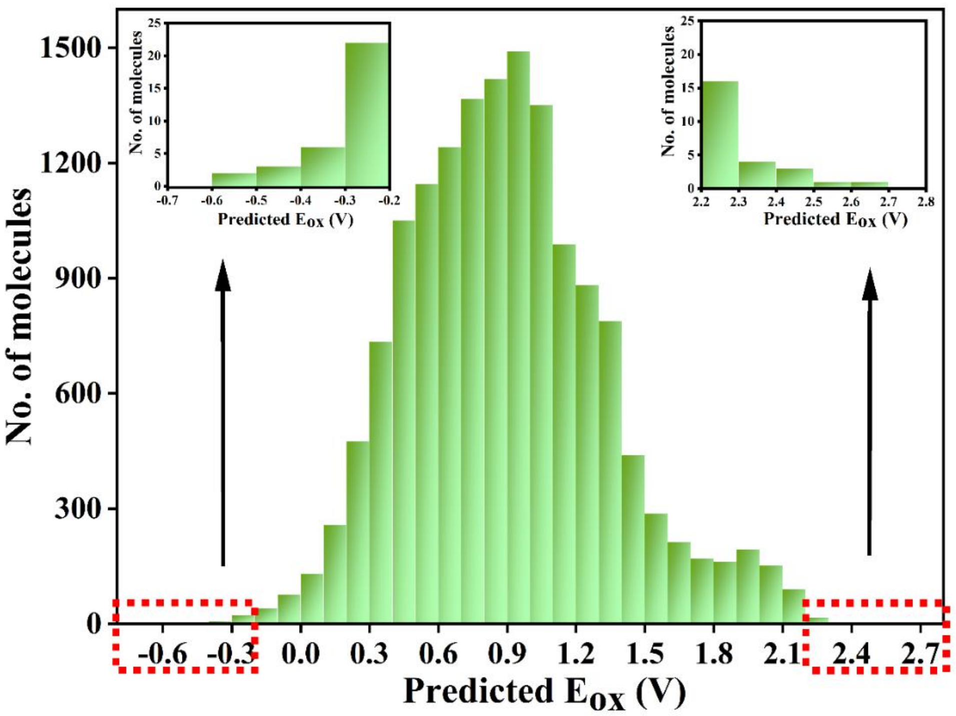

Fig. S1 E_{ox} distribution range of molecules incorporated in OxPot.

Table S1. Experimental and predicted E_{ox} values of the tested molecules.

Sr. No.	PubChem CID	SMILES	Experimental (V)	Predicted (V)	Difference
1.	6514	C(CS)C(=O)O	1.39	1.380	0.01
2.	66345	CNC1=CC=C(C=C1)C(=O)O	0.719	0.777	-0.058
3.	978	O=C(O)c1ccc(N)cc1	0.932	0.906	0.026
4.	403	C1=CC(=CC=C1N)O	0.454	0.431	0.023
5.	6115	C1=CC=C(C=C1)N	0.714	0.767	-0.053
6.	243	C1=CC=C(C=C1)C(=O)O	1.792	1.711	0.081
7.	7504	c1ccc(cc1)CN	1.328	1.365	-0.037
8.	8784	C1=CC=C(C=C1)C=CC(=O)O	1.27	1.290	-0.02
9.	81531	CCN(C(C)C)C(C)C	0.698	0.608	0.09
10.	674	CNC	1.261	1.166	0.095
11.	7935	C1=CC(=CC(=C1)N)N	0.651	0.573	0.078
12.	7243	C1=CC=C(C(=C1)N)N	0.354	0.447	-0.093
13.	7814	C1=CC(=CC=C1N)N	0.222	0.197	0.025
14.	2723949	S=C(N)C	1.180	1.216	-0.036
15.	1133	C(C(=O)O)S	1.399	1.493	-0.094
16.	273790	C(=S)(N)N	1.354	1.280	0.074
17.	8471	CCN(CC)CC	0.882	0.959	-0.077

Table S2. Linear correlation of experimentally measured E_{ox} and computationally calculated E_{HOMO} using various functionals

Basis set	Function	R^2	MAE	RMSE	Slope	Intercept
cc-pVDZ	PBE0	0.977117381	0.056948694	0.06405773	-0.661297047	-2.772900991
cc-pVDZ	X3LYP5	0.976069289	0.057552843	0.06550832	-0.671099236	-2.595116361
cc-pVDZ	X3LYP	0.976058188	0.057562602	0.06552351	-0.671292828	-2.639377275
cc-pVDZ	B3LYP	0.9757064	0.057578711	0.06600314	-0.675439392	-2.640124261
6-311G**	PBE0	0.975547407	0.057908268	0.06621877	-0.66400039	-2.844113818
6-311G**	B3LYP	0.974140053	0.057833423	0.0680977	-0.675773366	-2.713983269
cc-pVTZ	PBE0	0.97378552	0.059571878	0.06856292	-0.669074137	-2.891389485
6-311G**	X3LYP	0.97371884	0.059049199	0.06865006	-0.670694656	-2.707010871
6-311G**	X3LYP5	0.973705571	0.059062088	0.06866739	-0.670328507	-2.662049234
def2-TZVP	PBE0	0.972296081	0.05990921	0.07048379	-0.675597332	-2.941466132
cc-pVTZ	B3LYP	0.971601357	0.059397127	0.07136207	-0.684097192	-2.780570358
cc-pVTZ	X3LYP5	0.971096677	0.060333949	0.07199338	-0.679380755	-2.731796011
cc-pVTZ	X3LYP	0.97108698	0.060321901	0.07200545	-0.679624261	-2.776626726
6-31G*	PBE0	0.970794851	0.060277263	0.0723683	-0.640522167	-2.653322054
def2-TZVP	X3LYP	0.969307619	0.062859495	0.07418805	-0.687223355	-2.8297325
def2-TZVP	X3LYP5	0.969306477	0.062872203	0.07418943	-0.686980419	-2.78448415
def2-TZVP	B3LYP	0.969290075	0.060865899	0.07420925	-0.690525265	-2.827306277
6-31G*	B3LYP	0.968439228	0.062346556	0.07523024	-0.647722232	-2.48443607
6-31G*	X3LYP	0.967958057	0.063354359	0.07580155	-0.643397859	-2.48193468
6-31G*	X3LYP5	0.967900785	0.063405737	0.07586926	-0.64308382	-2.438805811
def2-TZVP	TPSS	0.944687681	0.077732449	0.09959318	-0.695984135	-2.270820423
cc-pvtz	TPSS	0.943193122	0.080300509	0.10092973	-0.687496673	-2.216306816
6-311G**	TPSS	0.936632747	0.085633412	0.1065985	-0.674118574	-2.130491564
6-31G*	TPSS	0.926771849	0.094427723	0.11459289	-0.643995818	-1.915379534
cc-pVDZ	TPSS	0.925660491	0.091695455	0.11545919	-0.664645655	-2.012191198
def2-TZVP	LDA	0.879988693	0.125302565	0.14669987	-0.683599304	-1.351261355
cc-pVTZ	LDA	0.875853101	0.125650891	0.14920611	-0.672027722	-1.295288446
6-311G**	LDA	0.867446848	0.125784298	0.1541749	-0.647128458	-1.182323773
6-31G*	LDA	0.864647773	0.125285981	0.15579423	-0.619174348	-0.96929675
cc-pVDZ	LDA	0.841989327	0.141101108	0.16833013	-0.636577828	-1.039915019

References

1. Méndez-Hernández, D. D.; Tarakeshwar, P.; Gust, D.; Moore, T. A.; Moore, A. L.; Mujica, V., Simple and accurate correlation of experimental redox potentials and DFT-calculated HOMO/LUMO energies of polycyclic aromatic hydrocarbons. *J. Mol. Model.* **2013**, 19, 2845-2848.
2. Marcus, R. A., On the Theory of Oxidation-Reduction Reactions Involving Electron Transfer. I. *J. Chem. Phys.* **1956**, 24, 966-978.
3. von Eschwege, K. G.; Conradie, J., Review of DFT-simulated and experimental electrochemistry properties of the polypyridyl Row-1 Mn, Fe & Co, and Group-8 Fe, Ru and Os MLCT complexes. *Electrochem. commun.* **2022**, 136, 107225.
4. Conradie, J., Reduction potential of benzophenones, hydroxyphenones and bis(2-hydroxyphenone)copper molecules. *Electrochim. Acta* **2023**, 443, 141931.
5. Conradie, J., Redox chemistry of bis(terpyridine)manganese(II) complexes – A molecular view. *J. Electroanal. Chem.* **2022**, 913, 116272.
6. D'Andrade, B. W.; Datta, S.; Forrest, S. R.; Djurovich, P.; Polikarpov, E.; Thompson, M. E., Relationship between the ionization and oxidation potentials of molecular organic semiconductors. *Org. Electron.* **2005**, 6, 11-20.
7. Landrum, G., *RDKit:* A software suite for cheminformatics, computational chemistry, and predictive modeling. 31.10 ed.; Greg Landrum: 2013; Vol. 8.
8. Cancès, E.; Maday, Y.; Stamm, B., Domain decomposition for implicit solvation models. *J. Chem. Phys.* **2013**, 139.
9. Sun, Q.; Zhang, X.; Banerjee, S.; Bao, P.; Barbry, M.; Blunt, N. S.; Bogdanov, N. A.; Booth, G. H.; Chen, J.; Cui, Z.-H.; Eriksen, J. J.; Gao, Y.; Guo, S.; Hermann, J.; Hermes, M. R.; Koh, K.; Koval, P.; Lehtola, S.; Li, Z.; Liu, J.; Mardirossian, N.; McClain, J. D.; Motta, M.; Mussard, B.; Pham, H. Q.; Pulkin, A.; Purwanto, W.; Robinson, P. J.; Ronca, E.; Sayfutyarova, E. R.; Scheurer, M.; Schurkus, H. F.; Smith, J. E. T.; Sun, C.; Sun, S.-N.; Upadhyay, S.; Wagner, L. K.; Wang, X.; White, A.; Whitfield, J. D.; Williamson, M. J.; Wouters, S.; Yang, J.; Yu, J. M.; Zhu, T.; Berkelbach, T. C.; Sharma, S.; Sokolov, A. Y.; Chan, G. K.-L., Recent developments in the PySCF program package. *J. Chem. Phys.* **2020**, 153.
10. Sorkun, M. C.; Koelman, J. M. V. A.; Er, S., Pushing the limits of solubility prediction via quality-oriented data selection. *iScience* **2021**, 24.
11. Sorkun, E.; Zhang, Q.; Khetan, A.; Sorkun, M. C.; Er, S., RedDB, a computational database of electroactive molecules for aqueous redox flow batteries. *Sci. Data* **2022**, 9, 718.
12. Sorkun, M. C.; Khetan, A.; Er, S., AqSolDB, a curated reference set of aqueous solubility and 2D descriptors for a diverse set of compounds. *Sci. Data* **2019**, 6, 143.
13. Huuskonen, J., Estimation of Aqueous Solubility for a Diverse Set of Organic Compounds Based on Molecular Topology. *J. Chem. Inf. Comput.* **2000**, 40, 773-777.
